# Health status of Native people living in the Republic of Sakha (Yakutia)

**DOI:** 10.3402/ijch.v72i0.21166

**Published:** 2013-08-05

**Authors:** Tatiana Burtseva, Tatiana Uvarova, Maya Savvina, Viktor Shadrin, Sergei Avrusin, Vyacheslav Chasnyk

**Affiliations:** Yakut Research Centre for Complex Medical Problems SB RAMS, Yakutsk, Russian Federation, Saint-Petersburg State Pediatric Medical Academy, Saint-Petersburg, Russian Federation

**Keywords:** indigenous people, examination, regions of Yakutia, prevalence of diseases, cross-sectional survey, standards of living

## Abstract

**Background:**

Native people of the Republic of Sakha (Yakutia) live mostly in northern regions in the so-called “national settlements”. Natives usually experience more health-related problems as compared to the total population. As a result, life expectancy at the birth of Natives living in the Republic of Sakha (Yakutia) is lower compared to ethnic groups living in European countries, in the United States, and in Canada.

**Objective of the study:**

To determine the prevalence of diseases among Natives living in Yakutia and to compare the standards of living for Dolgans living in the Anabarsky region and Evenks living in Gigansky and Ust-Maysky regions.

**Study design/methods:**

The study was designed as a population-based, cross-sectional examination with the addition of a cross-sectional survey for Dolgans and Evenks. Data were obtained from 324 Evenks, 43 Evens, 230 Dolgans, and 216 people of other ethnic groups, aged 17–86. In the additional cross-sectional survey, 155 Dolgans and 292 Evenks were included.

**Results:**

Among Natives, the most prevalent diseases are digestive diseases (67.9 cases per 100 examined), diseases of the genitourinary system (45.3 per 100 examined), circulatory system diseases (44.4 per 100 examined), diseases of the respiratory system (36.9 cases per 100 examined) and diseases of the musculoskeletal system and connective tissue (28.4 cases per 100 examined). There are differences in the prevalence among Natives living in different regions. Anabarsky region has the lowest disease burden and Dolgans inhabiting this region have higher standards of living than Evenks living in Gigansky and Ust-Maysky regions.

**Conclusions:**

The prevalence of diseases among the Natives, living in the Republic of Sakha (Yakutia), is very high. Differences in raw prevalence rate between Native ethnic groups were found, but it is unknown whether these differences can be assigned rather to the difference in standards of living in the inhabited locality than to ethnicity itself.

Indigenous people living in the Republic of Sakha (Yakutia), who are jointly called Natives, can be divided into 5 major ethnic groups: Evens, Evenks, Yukagirs, Chukchi and Dolgans belonging to 4 language families (Fig. 1). This grouping is based on cultural and linguistic similarities of people living in different—mostly northern—regions of the Republic of Sakha (Yakutia) mainly in the so-called “national settlements” where the number of Native families as a rule is more than 60%. Lifestyle, labour activity and food preferences of the Natives, being dependent to some extent on the ethnicity of the community, is mostly influenced by the environment (tundra, forest, sea shore, bank of a river).

**Fig. 1:**
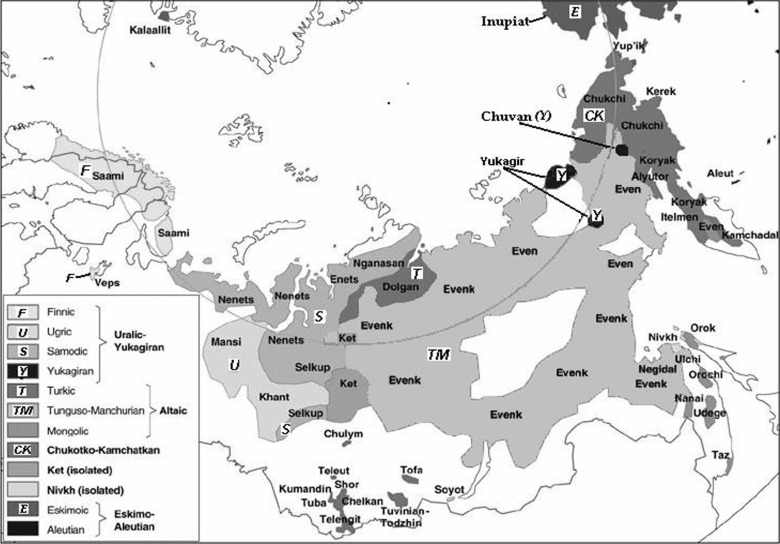
Indigenous peoples of the North, Siberia and Far East of the Russian Federation, subdivided according to language families. Polar Circle is marked with grey line (1).

Indigenous people usually experience more health related problems as compared to the total population (2–5). Their health status and outcomes are embedded within the specific socio-economic, political and cultural contexts, as well as influenced by ecology issues. It is also known that the number of consanguineous marriages in Yakutia, including first-cousin unions, is rather high (6).

As a result, life expectancy at birth of Natives living in the northern European, Far Eastern regions of the Russian Federation and in Siberia is significantly lower compared to ethnic groups living in European countries, US, and Canada (7).

Objective of this study is to determine the prevalence of diseases among Natives living in Yakutia and to compare the standards of living for Dolgans living in the Anabarsky region and Evenks living in Gigansky and Ust-Maysky regions.

## Materials and methods

The study was designed as a population-based, cross-sectional examination, based on the results of the longitudinal analysis of national and regional reports of the Yakut Healthcare services, performed according to the standard diagnostic programs with the addition of a cross-sectional survey for Dolgans and Evenks.

Analysis included 813 people (547 women—67.3%; 266 men—32.7%) aged 17–86 years (average 44.0±1.7 years), living in Gigansky, Ust-Maysky and Anabarsky regions of Yakutia. The following were included: Evenk—324 (40%), Even—43 (5%), Dolgans—230 (28%), others—216 (27%). Examinations were performed by a group of medical specialists including cardiologists, pulmonologists, neurologists, gastroenterologists, ENT-specialists, urologists, gynaecologists, endoscopists and specialists in medical ultrasonography.

In the additional cross-sectional survey, a total of 447 Natives were surveyed (155 Dolgans and 292 Evenks).

To determine the significance of differences, a 2-sample *t*-test was used. To determine the significance of the difference between percentages, a 2-sample *t*-test between percentages (independent samples) was used (Statistics calculator, StatPac, ver. 4).

## Results

It was found that among Natives living in the Republic of Sakha (Yakutia), the most prevalent diseases, on average, are digestive diseases (67.9 cases per 100 examined), diseases of the genitourinary system (45.3 per 100 examined), circulatory system diseases (44.4 per 100 examined), diseases of the respiratory system (36.9 cases per 100 examined) and diseases of the musculoskeletal system and connective tissue (28.4 cases per 100 examined) (Table I).

**Table I:** Native people of Yakutia: average prevalence of diseases in terms of the International Statistical Classification Of Diseases And Related Health Problems 10th Revision

Class ICD 10 (version: 2010)	Per 100 examined
Certain infectious and parasitic diseases (A00-B99)	4.4
Neoplasms (C00-D48)	2.8
Endocrine, nutritional and metabolic diseases (E00-E90)	8.7
Mental and behavioral disorders (F00-F99)	0.6
Diseases of the nervous system (G00-G99)	15.0
Diseases of the ear and mastoid process (H60-H95)	3.0
Diseases of the circulatory system (I00-I99)	44.4
Diseases of the respiratory system (J00-J99)	36.9
Diseases of the digestive system (K00-K93)	67.9
Diseases of the musculoskeletal system and connective tissue (M00-M99)	28.4
Diseases of the genitourinary system (N00-N99)	45.3
Congenital malformations, deformations and chromosomal abnormalities (Q00-Q99)	2.6
Injury, poisoning and certain other consequences of external causes (S00-T98)	1.2
Total	261.2

Selected results of comparative analysis of the prevalence of the most common diseases in Ust-Maysky, Gigansky and Anabarsky regions of the Republic of Sakha (Yakutia) are presented in Table II. Presented data show that there are somewhat great differences in the prevalence among Natives living in different regions; the Anabarsky region appears to have the lowest disease burden.

**Table II:** Native people of Yakutia: average prevalence of diseases in different regions in terms of the International Statistical Classification of Diseases and Related Health Problems 10th Revision

	Regions of Yakutia					
	
	Ust-Maysky		Gigansky		Anabarsky	
			
Class ICD 10 (version: 2010)	Number of cases	Per 100 examined	Number of cases	Per 100 examined	Number of cases	Per 100 examined
Diseases of the circulatory system (I00-I99)	103	55.4	97	51.3	161	36.8
Diseases of the respiratory system (J00-J99)	75	40.3	96	50.8	129	29.5
Diseases of the digestive system (K00-K93)	139	74.7	148	78.3	265	60.1
Diseases of the musculoskeletal system and connective tissue (M00-M99)	77	41.4	55	29.1	99	22.6
Diseases of the genitourinary system (N00-N99)	79	42.5	109	57.7	180	41.1

**Fig. 2:**
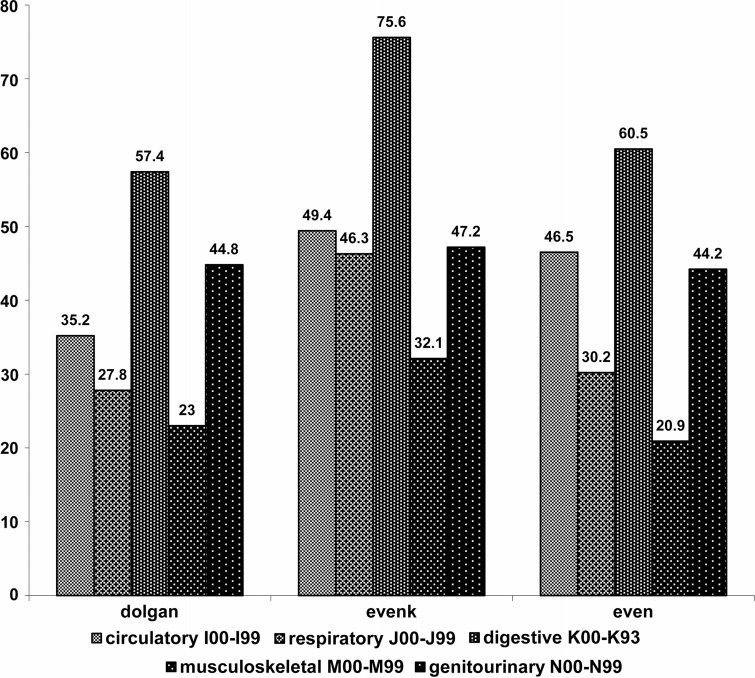
Native people of Yakutia: prevalence of diseases (per 100 examined) in different ethnic groups in terms of the International Statistical Classification of Diseases and Related Health Problems 10th Revision.

The results of comparative analysis of the prevalence of the most common diseases in subpopulations of Evens, Evenks and Dolgans—the main studied ethnic groups—are shown in Fig. 2. Although differences can be seen, there are not enough data to distinguish the differences related to ethnicity and to inhabited locality separately.

Selected results of the survey analysis are shown in Table III. Comparison of characteristics No. 2–6, presented in Table III, shows that the standards of living for Evenk people are significantly lower than that for Dolgan people.

**Table III:** Selected results of the survey analysis

		Ethnic group		
		
#	Parameter	Dolgans	Evenks	P=
1	Average square meters for an apartment	13.9	18.7	0.180
2	Central heating system (% of surveyed)	95.5	34.9	0.000
3	House built after the year 1995 (% of surveyed)	45.8	28.1	0.000
4	Quality of apartment below the current standards of suitability (% of surveyed)	21.3	40.1	0.000
5	Average income per capita (roubles)	8372	5675	0.001
6	Income below or at the level of the current lower living standards (%)	31.0	40.4	0.050

## Conclusions

The prevalence of diseases among the Natives living in the Republic of Sakha (Yakutia) is very high specifically for digestive diseases, diseases of the genitourinary system, circulatory system, respiratory system, musculoskeletal system and connective tissue.

Statistically significant differences in raw prevalence rates between Native ethnic groups were found, but it is unknown whether these differences can be assigned to the difference in standards of living in the inhabited locality rather than to ethnicity itself.

Standard of living for Dolgans who live in Anabarsky region is significantly higher compared with the standard of living indicators for Evenks inhabiting Gigansky and Ust-Maysky regions of the Republic of Sakha (Yakutia).

The results of the study are the basis for assessment of disease burden in subpopulations of Natives and also for the individualization of health care programs.
